# Perceived barriers in accessing food among recent Latin American immigrants in Toronto

**DOI:** 10.1186/1475-9276-12-1

**Published:** 2013-01-03

**Authors:** Mandana Vahabi, Cynthia Damba

**Affiliations:** 1 Faculty of Community Services-Daphne Cockwell School of Nursing, Ryerson University, 350 Victoria Street, Toronto, ON, M5B 2k3, Canada; 2 Centre for Studies in Food Security, Ryerson University, 350 Victoria Street, Toronto, ON, M5B 2k3, Canada; 3 Epidemiologist, Toronto, ON, Canada

**Keywords:** Canada-Toronto, Recent Latin American immigrants, Food security, Cultural and Linguistic barriers, Community- based food programs

## Abstract

**Objective:**

In Canada, recent immigrant households experience more food insecurity than the general population, but limited information is available about the personal, cultural, and social factors that contribute to this vulnerability. This study focused on recent Latin American (LA) immigrants to explore their perceived barriers in acquiring safe, nutritious, and culturally-appropriate food.

**Design:**

A cross-sectional mixed-method design was applied to collect information from a convenience sample of 70 adult Spanish/Portuguese speakers who had arrived in Toronto within the last five years. Face-to-face interviews were conducted with primary household caregivers to obtain responses about barriers to acquiring food for their households; data were analyzed using a thematic analysis technique.

**Results:**

Four main categories of barriers were identified: limited financial resources; language difficulty; cultural food preferences; and poor knowledge of available community-based food resources and services. Inadequate income was the main impediment in accessing adequate food, and was related to affordability of food items, accessibility of food outlets and transportation cost, and limited time for grocery shopping due to work conditions. Language barriers affected participants’ ability to obtain well-paid employment and their awareness about and access to available community-based food resources. Cultural barriers were related to food preferences and limited access to culturally-appropriate foods and resources.

**Conclusion:**

The main barrier to food security among our sample of LA newcomers to Toronto is limited financial resources, highlighting the need for policies and strategies that could improve their financial power to purchase sufficient, nutritious, and culturally-acceptable food. Linguistic barriers and limited information among newcomers suggest the need to provide linguistically- and culturally-appropriate information related to community-based food programs and resources, as well as accessible subsidized English language programs, in the community and at workplaces. Participatory community-based food programs can augment, in a socially acceptable manner, food resources and reduce the social stigma attached to food charity. Finally, it is crucial to monitor and evaluate existing social and community-based services for their accessibility, cultural appropriateness and diversity, and effectiveness.

## Introduction

Immigrants face many challenges including unemployment, underemployment, lack of affordable and safe housing, linguistic barriers, and limited social capital [1-6]. These cumulative disadvantages may make immigrants more vulnerable to food insecurity and its related health problems, but little is known about the personal, cultural, social, and economic factors that contribute to recent immigrants’ household food security.

The increasing numbers and diversity of immigrants in Canada and the persistently higher prevalence of food insecurity among newcomers, in particular Latin Americans [7,8], necessitate a close examination of recent immigrants’ experiences and challenges in accessing safe, nutritious, and culturally-acceptable food. This paper reports the findings from a cross-sectional mixed-methods exploratory study of recent Latin American (LA) immigrants’ perceived barriers in accessing food in Toronto.

## Literature review

Food is a fundamental human need and limited access to food is considered a violation of human rights [9]. Food security is defined as a condition that exists when all people, at all times, have physical and economic access to sufficient, safe, and nutritious food to meet their dietary needs and food preferences for an active and healthy life [10,11]. Household food insecurity (HFI) implies limited ability or uncertainty in accessing adequate, nutritious, and culturally-acceptable food in socially-acceptable manners [12]. Socio-economic determinants of food security include, but are not limited to, income, source of income, housing circumstances, other household costs (e.g., utilities, repairs, childcare, remittances back to the home country), social capital, and neighbourhood food access [13-18].

Food security is a significant social determinant of health [19]. Ample evidence suggests a direct relationship between household food security and physical, mental, and social health [18,20-24]. Food-insecure individuals are more likely to have one or more chronic illnesses such as type 2 diabetes or heart disease; to experience mental illnesses such as depression; and to be more prone to obesity or overweight [13,18,25,26]. Moreover, food-insecure individuals are more vulnerable to feelings of anxiety, powerlessness, worthlessness, and family dysfunction stemming from a preoccupation with obtaining food through socially-stigmatized means such as food banks, borrowing money, selling belongings, or stealing [21,27,28]; reliance on such means to obtain food can also lead to increased debt and social deprivation [21].

The prevalence of food insecurity in Canada has gradually been decreasing over the past decade, but remains high among recent immigrants [7]. Although the food insecurity prevalence rates for recent immigrants in Canada, especially for Latin Americans, are significantly higher than national and provincial averages, there is limited research on the personal, cultural, and social factors that contribute to such vulnerability among this population.

According to the most recent available census data (2006) [29], one in five Canadians is foreign-born, and 84% of immigrants who arrived from 2001–2006 were born in non-European countries, often those affected by war or social, political, and economic unrest. From 2001–2006, the visible minority population grew almost five times faster than the total population (27.2% vs. 5.4%).

This paper focuses on Latin American (LA) immigrants in Toronto, Canada. LAs are a fast-growing and relatively new visible minority from non-European countries in Canada. They are a heterogeneous group from North, Central, and South America, generally with Spanish or Portuguese as a first language. From 2001–2006, the LA population in Canada increased by 40% to more than 304,000. According to the 2007–2008 Canadian Community Health Survey (CCHS) [7], LA recent immigrant households were among the top recent immigrant groups facing food insecurity (45.7%), but this rate may underestimate the true prevalence of food insecurity among this group because the sampling strategy does not consider the Canadian ethno-racial composition.

Like other immigrant groups to Canada, many LA immigrants are relatively well-educated, but most have low incomes due to difficulty in securing jobs that match their education and skills. According to Statistics Canada [8], LA immigrants had higher rates of unemployment (8.5% vs. 6.7% among the general population), lower average incomes ($26,354 vs. $40,704), and more low-income families. Inability to speak English or French also reduces their low job prospects and increases other challenges related to acculturation [30,31].

Several studies have focused on food insecurity among LA immigrants in the United States [32,33], but few have explored this issue in Canada. Most studies have focused on immigrants in general or on the subset using food banks [31,34]. This study is among the first few to explore perceived barriers to accessing safe, nutritious, and culturally-acceptable food among LA immigrants to Canada.

## Methods

We conducted a cross-sectional mixed-methods exploratory study to assess the prevalence of food insecurity, its correlates, dietary intake, and perceived barriers to accessing food among recent LA immigrants residing in Toronto. This paper focuses on qualitative findings related to the perceived barriers to accessing safe, nutritious, and culturally-acceptable food.

### Sample

There is no available sampling frame for this population and the proportion of food insecurity among the LAs is unknown. Considering that this was an exploratory study, we recruited a purposive sample of 70 adult LAs using convenience (from selected community health centres (CHCs) in Toronto) and snowball sampling strategies [35], which increased the possibility of recruiting recent immigrants who are frequently difficult to reach. A sample of 70 also allowed for preliminary exploration of correlation among variables in our quantitative phase of the study. The target population included adults who: 1) were Spanish/Portuguese speakers from Central or South America; 2) were 20 years or older; 3) were primary caregivers in charge of household expenses including food purchase; and 4) had immigrated to Canada within the past five years. The study was advertised through posters at selected CHCs; health care professionals at CHCs also referred eligible subjects to the research assistants (RAs), who contacted participants, assessed eligibility, and set a convenient time and place for interviews. Participants received a $10 honorarium.

#### Data collection and measures

The Human Subjects Review Committee at Ryerson University approved the study protocol. Data were collected from June–October 2008 through face-to-face interviews conducted in the participant’s native language (Spanish or Portuguese) by bilingual RAs. Participants were informed about their rights, including voluntary participation or withdrawal at any time. Written consent was obtained from all participants. Most interviews took place in participants’ homes, with the exception of a few which were held in a private room at the CHCs where the participants visited. Interviews involved three questionnaires and took approximately one hour to complete. The study questionnaires were translated into Spanish and Portuguese and then back-translated into English by two independent bilingual researchers. The translated version was pilot-tested for flow, clarity, and comprehension through personal interviews with five members of the LA community. The first questionnaire collected self-reported information about socio-demographic and self-reported health characteristics (e.g., age, sex, marital status, education, household income, length of stay in Canada, number of people in the household, type of dwelling, knowledge of official languages, self reported health status, presence of chronic health conditions including mental health and stress levels before and after immigration), which were extracted from standard questionnaires used in the 2006 Canadian Census and the national Canadian Community Health survey (CCHS 2.2); the second measured food insecurity using the Spanish and Portuguese version of the 18-item CCHS 2.2 Food Security Module, which classifies households during the previous 12 months as “food secure,” “food insecure-moderate,” and “food insecure-severe” (see Table 1); and the third assessed self-reported dietary intake within the last 24 hours using Canadian food Guidelines, use of health, social, and community services including food banks, and perceived barriers in accessing food. This paper mainly focuses on the findings from the open-ended question about barriers to food acquisition: “In your opinion, what have been the major barriers or facilitators in accessing the required food that you need for yourself and your family?” The results regarding the extent and correlates of food insecurity among LA immigrants residing in Toronto were published in Journal of Immigrant and Minority Health, 2011 (for more details please see reference # [17]).

**Table 1:** Health Canada food security category specification

**Category labels**	**Category description**		
	**10-item adult food security scale**	**8-item child food security scale**	**Household food security for household with children**
**Food Secure**	0-1 affirmed responses indicating difficulty with income-related food access	0-1 affirmed responses	Both adults and children food secure
**Food Insecure, Moderate**	2-5 affirmed responses indicating compromise in quality and/or quantity of food consumed	2-4 affirmed responses	Either adults or children (or both) food insecure, neither severely food insecure
**Food Insecure, Severe**	≥ 6 affirmed responses indicating reduced food intake and disrupted eating patterns	≥ 5 affirmed responses	Either adults or children (or both) severely food insecure

We decided not to audio-tape interviews because we were concerned that it might deter participants from expressing themselves freely. Although we did not ask about immigration status, audio-taping may have caused some participants to be fearful of deportation and to view the study as too risky. Therefore, to ensure accurate recording of responses to the one open-ended question RAs captured the comments verbatim, read them back to the respondent, and adjusted as necessary. The RAs probed further only when the responses were not clear and required further clarification. All the participants shared their experiences and challenges in accessing food since their arrival to Canada and their responses ranged from a few lines to a page. Together, the responses generated approximately 51 pages of data, which RAs translated into English.

This paper discusses socio-demographic and food security status of participants in relation to findings from an the open-ended question about barriers to food acquisition mentioned above. 

## Data analysis

Socio-demographic data were analyzed using the Statistical Package for Social Sciences (SPSS) version 16 for Windows. Descriptive statistics were used to summarize socio-demographic and self-reported health characteristics. RAs translated responses to the open-ended question, and the translated transcripts were analyzed by two research team members (M.V & C.D) using an inductive analysis technique, which allows relevant themes and categories to emerge from transcribed interviews [36]. Analysis involved systematic reading of the text, highlighting important passages and words, and organizing these into preliminary codes or category schemes. Further interpretation and modification of coding was conducted after rereading the text sorted using the preliminary codes and checking for disconfirming evidence [36]. Inconsistencies were reviewed and discussed to achieve consensus, and the integrity of data interpretation was maintained using strategies of credibility (peer debriefing), and authenticity (use of direct quotes). We analysed the qualitative data in light of the participants’ food security status and highlighted the barriers and themes that were common and/or different according to food security status, gender, and presence of household members with chronic diseases.

## Results

### Socio-demographic characteristics

Table 2 presents socio-demographic characteristics of the 70 participants. The largest fraction came from Colombia, followed by Brazil and Mexico. Most were young women, and nearly two-thirds were married or in common-law relationships. Although most were fairly well educated, many had arrived less than a year earlier and more than half could not speak English well.

**Table 2:** Socio-demographic characteristics and self-reported health status (n = 70)

**Sex**	***#***	***%***
Male	13	17.6
Female	57	82.4
**Age (years)**	***Median (SD)***	***Range***
	33 (8.8)	20-56
		**%**
20-29		33
30-39		32
40-56		33
**Country of Origin**	***#***	***%***
Brazil	20	28.6
Colombia	30	42.9
Mexico	20	28.6
**Marital status**	***#***	***%***
Married (including common -law)	46	65.7
Single (including single, separated, divorced, widowed)	24	34.3
**Highest Education Level Completed**	***#***	***%***
Secondary school diploma or equivalent	15	21.4
Apprenticeship/trade/college/other non-university	23	32.9
University degree or higher	32	45.7
**Family size**	**#**	**%**
1	17	24.3
2-3	27	38.6
4 or more	26	37.1
Mean (SD )	3 (1.45)	
Range	1-7	
**Number of children under 18**	***#***	***%***
0	32	45.7
1	18	25.7
2	16	22.9
3 +	4	5.7
**Households with children under 6**	**#**	**%**
No children under 6	43	61.4
Children under 6 years old	27	38.6
**Time in Canada**	***#***	***%***
< 1 yr	31	44.3
1-5 yrs	39	55.7
**Fluency in English**	**#**	**%**
Poor	39	55.7
Good	27	38.6
Excellent	4	5.7
**Household average monthly income ($)**	***#***	***%***
< $1,500	28	40
$1,500 - $ 1,999	12	17.1
> $2,000	27	38.6
Don't know	3	4.3
**Main source of income (past 12 months)**	***#***	***%***
Wage/salary	36	51.4
Provincial/municipal assistance/welfare	33	47.1
Self-employment	18	25.7
Child tax benefit	14	20
Child support	10	14.3
Other (including employment insurance and retirement pensions)	7	10
**Stress level most days since arrival in Canada**		
Extremely stressful/Quite stressful	16	22.9
Somewhat stressful/Slightly stressful	32	45.8
Not stressful	22	31.4

Nearly one-quarter of households were single-person households and 39% included between two and three individuals. More than half of all households had included between one and four children under 18 years, and 39% had children younger than six years. More than half of all households included individuals with one or more chronic conditions: depression was the most common, followed by intestinal or stomach disorders and high blood pressure. Household income was fairly low; almost half reported incomes less than CAD $1,500/month. Approximately half were receiving welfare, but the main source of income was salaries/wages from full- or part-time employment. Almost 90% lived in rental housing, and most spent more than half their income on rent. Two-thirds used food banks. The prevalence of food insecurity increased with decreasing income level: three-quarters of households earning less than $1,500/month were food insecure, compared with one-third of those earning more than $1,500/month. Overall, food insecurity rates were high: less than half were food secure and the rest were moderately or severely food insecure. Households receiving welfare were significantly more likely to be food insecure than others, as were households using food banks.

### Barriers to food acquisition

We identified both structural and socio-economic barriers to food acquisition. These fell into four main categories: limited financial resources, limited/poor language proficiency, limited access to culturally preferred food, and poor knowledge of community-based food resources and services. The majority of respondents identified several barriers to food acquisition. We observed differences in types and levels of barriers based on participants’ food security status, gender, particularly for food insecure mothers, and single mothers, and presence of chronic diseases. Some respondents who were food secure also reported barriers that they had experienced earlier on in the process of their immigration. Throughout the interviews, psychosocial ramifications such as depression, anxiety, the feeling of shame, low self-esteem, and powerlessness related to the four barriers were also expressed by respondents.

#### Limited financial resources

Inadequate income was identified as the main impediment to accessing adequate food, particularly for those respondents who were food insecure. Insufficient income was related to difficulty finding employment or having low-paying jobs such as waitressing, babysitting, or cleaning; this was despite the fact that many participants were well-educated and had extensive work experience in their country of origin. Their difficulties were often related to language barriers and their qualifications not being recognized in Canada.

"I do not have enough money to buy what we need. I cannot get a job which suits my training. I work odd jobs just to pay the bills and put food on the table. But the pay is not sometimes enough for everything which needs to be paid."

The seasonal nature of some work, like construction, also meant that participants were unemployed during the long Canadian winter. This was a common barrier for those who were food insecure.

"I do construction work whenever I could get it. The pay is better but it slows down in winter time and then I have difficulty in paying bills and getting food."

Due to inadequate income, many participants were forced to rely on welfare. However, participants with unstable immigration status refrained from applying for assistance for fear of being discovered and deported. Of those receiving welfare, many indicated that it was insufficient to cover high costs of housing and food. This was true for several of those participants who were food insecure.

"Although we get welfare the rent is very expensive. We cannot find cheaper houses here or anywhere else. Otherwise it would be easier to get nutritious food for my kids."

It was even more difficult for participants with a physical disability to find work. Most participants noted the lack of government support to newcomers to help them establish private businesses. Some had left their home country believing they could find good jobs in Canada that did not materialize, forcing them to work at whatever job was available.

"I feel cheated and cannot do anything about it. All those interview and documentation to enter to Canada meant nothing. I sometimes feel I should get back. I did not know what hunger meant until I got here."

Costs associated with transportation posed another barrier to food access; inexpensive grocery stores were often far from participants’ residences and thereby inaccessible without a car or public transit, especially in winter. This issue was mainly raised by respondents who were food insecure.

"The job pays little and near our place they are not that many big food stores like No Frills [A large discount supermarket]. We do not have a car and have to take the TTC [Toronto Transit Commission] for transportation and that is costly when you have to pay for rent and other bills."

Inadequate income also affected food security by restricting food purchases in terms of quantity and quality. Respondents who were food insecure focused on food quantity and quality whereas those who were food secure mainly talked about food quality. The issue of inadequate quantity of food and poor/less desirable quality of food was compounded by the seasonal availability of some foods, which makes them hard to find and more expensive in winter.

"Foods are more expensive in Canada, especially fruits and vegetables compared to Mexico. We are used to eating fresh food and here we find it impossible."

"To stay within our budget I do not buy fresh vegetables or fruits anymore. Here we eat either frozen food or out of a can because those are cheap and we can afford."

Use of food banks was reported mainly by respondents from food insecure households. However, a few respondents who were food secure also used food banks. Frequency varied from one to four times a month. It was not clear whether frequency of use was dictated by food bank regulations or individual need. Many of the participants who did use food banks found the food to be low-quality, culturally unacceptable, and insufficient:

"They offer limited variety of foods and they do not cater for those who have health conditions and require special diets. Almost everything is canned and sometimes we do not know what we are taking home. You cannot get fresh food and if you are lucky to get them they are almost rotten."

"We sometimes eat one meal a day as we are a family of five and we only get food from the food bank - “toca aguantar” [what can one do?]x"

Access to food banks was also limited for some, e.g., participants with unstable immigration status could not use food banks that requested identification documentation. In addition, food bank hours sometimes overlapped with school or work hours, making it difficult for some to get food: *“I do not get there soon enough to get food.”*

#### Health impact of food insecurity

Participants’ responses revealed physical and psychological stresses resulting from inadequate income. Some worried that dependency on high calorie and high fat foods might result in health problems such as becoming overweight; a few mentioned that they had gained weight since coming to Canada. Many mothers worried about not providing enough nutrients for their growing children:

"My children are growing and they need food to keep their strengths. I try to give them meat from time to time but that is not enough. Sometime if we have some extra money I give them fresh vegetables with cooked meat. I am concerned about their health. I hate myself when I give them junk food rather than real food."

"I haven’t got enough money to buy infant formula for my kid … I am quite worried about my children. They are not getting what they should. I feel useless."

Insufficient income particularly affected women, single mothers, pregnant women, and mothers with infants. Women appeared to jeopardize their food intake to benefit their children’s and partners’ dietary intake. As one woman stated:

"Often when there is not enough food for everyone I make sure that my children and husband are fed first. Those are the priorities. I still am concerned about not providing enough nutrients for my growing children."

Another group that was particularly affected were individuals requiring special diets (e.g., to manage chronic diseases like diabetes or food allergies/sensitivities such as lactose intolerance). People with chronic diseases were unable to afford foods specified for their health, avoid inexpensive but unhealthy options, eat at recommended times due to long shift work schedules; some could not afford to buy supplements for their condition such as calcium for osteoporosis.

Many participants resorted to obtaining food from food banks, family/friends, or institutions such as churches. However, this was complicated by stigma; inability to provide for ones’ family was culturally unacceptable for some participants. Some were so ashamed that they refused to use food banks or ask for help from friends/family:

"Every time I go to food bank I make sure that no one who I know sees me there. I feel ashamed and undignified. Thanks God my children are small and do not understand what is happening here. My family back home do not know how we live like this."

The constant worry about money and the related aspects of having to adjust to lower social positions made them depressed.

#### Language barriers

Language barriers were another common problem identified by many respondents who were food insecure. A few respondents who were food secure also mentioned they had language problems currently, or they had them previously, which had impeded their ability to be food secure at the time. Many participants could not speak English or French well, which led to problems obtaining food. One participant stated,

"My English is not so good. I have difficulty reading the food labels and communicating to shop keeper what I need when purchasing food."

Others noted that labels were not clear or easily interpretable and that they could not understand the ingredients and nutritional composition of foods, making it difficult to evaluate the quality and health consequences of the foods.

Language barriers also restricted shopping choices. Some participants said that it was more difficult to shop in small grocery/convenience stores where they had to ask the storekeeper for food, rather than large chain stores where they could search for food on their own. One participant said,

"Language has been a challenge. I want to ask for certain foods at the store but I do not know the words."

Language barriers also prevented participants from benefiting from food-related information from media and printed materials (newspaper flyers, discount coupons):

"I do not know where to get the discount coupons for foods I purchase or where or what is on sale. Sometimes my friends take me there but most time we miss them. I wish those announcements were offered also in Spanish."

Beyond the practical limitations of language barriers for food access, some participants, mainly women, noted that not being able to communicate while grocery shopping contributed greatly to their social exclusion and depression:

"Back home, grocery shopping is a daily routine that offer the opportunity for social interaction with other people. Here I feel isolated and depressed as I do not speak the language and cannot go out to talk to others."

Although many participants were enrolled in English as a Second Language classes, such programs were perceived to be limited by lack of advanced English language opportunities or opportunities for practice.

#### Cultural food preferences

Another barrier was related to cultural food preference and the lack of culturally-appropriate resources. As indicated earlier food security does not only mean having enough food but also ability to freely access culturally preferred food. Vegetables, fruits, and meats that were common in participants’ home countries were often hard to find and expensive in Canada:

"I have tried to look for foods that I am used to back home, but I have not been lucky. I would like to eat fruits such as guava, papaya, nopales (prickly pear), icama (Mexican potato). We now mainly eat cereals, pasta, rice, and legumes."

Many participants also said that the quality, taste, and smell of some foods differed from those in their home countries. Although both food secure and food insecure participants raised this issue of quality of food, this was one of the main barriers cited by the former group. Food secure people’s main complaint was that foods in Canada do not taste the same as their native countries. In particular, many said that fruits, meat, and cheese were bland and they associated the poor taste with poor nutritional quality.

Some participants considered Canadian food habits unhealthy and complained that many foods were “*too fatty/oily*,” *“too sweet,” “too spicy,”* or *“too salty*.” They indicated that frozen and fast foods (which are readily available and often more affordable) had low nutritional value and were potential health hazards, especially for young children.

"All the ready-to-eat food I found to be unhealthy here. The frozen foods are salty and full of preservative and dye. Fast food (is) too oily and nothing is fresh about the meat … In Colombia we cook everything from scratch. My mom goes shopping every day, buys everything fresh and then cooks them. It is really something new here and you have to adjust."

Participants noted that few stores carried ethnic foods in some neighbourhoods, and explained that these stores helped them stay “*connected to home type food*.” One participant stated,

"It is nice to see that some larger food chain stores like “No Frills” carry some of our ethnic food at reasonable prices. I hope other stores do the same."

Many respondents felt that most community-based resources like food banks offered culturally-inappropriate and limited foods, mainly canned and dried foods like bread, pasta, and canned tomatoes that some participants did not know how to prepare. Similar comments were voiced by some participants who ate at shelters.

Some participants had adjusted their preferences and had started eating some Canadian foods, which were more available and cheaper. Several food secure respondents mentioned that they took advantage of the readily available frozen foods.

#### Poor knowledge of community-based food resources and services

Limited information about community-based food resources was another common barrier to food security. Some participants did not know where to obtain cheaper foods or specific foods (ethnic, organic) at reasonable prices. This issue was raised mainly by those who were food insecure. This was especially true in the first years in Canada as stated by one of the food secure participant:

"It took us two years to find out that the foods we wanted such as granola and other grains could be purchased in bulk from bulk food stores at reasonable prices. I wish we had some information beforehand."

Some participants were unaware of Canadian support programs such as welfare, child supplements, and food banks, because these were not available in their home countries:

"Our knowledge is limited about any of the programs and services that are available in Canada because none of them exist back home. It would be good to get a list of available resources…then we know what to do or where to go."

Another problem (raised mainly by food insecure participants) was related to misinformation about available resources. Information was often provided by family/friends, other community members, and community and government agencies, and the different sources sometimes provided conflicting information. One respondent said, 

"the information varies depending on who you talk to. So at the end you do not really know if you are eligible or not."

## Discussion

This study is among the first few studies that explore barriers in accessing food among Canadian recent LA immigrants residing in Toronto. We found that these immigrants face both economic and non-economic challenges that may affect their food security. There were differences in the type and impact of challenges noted by level of food security, certain subgroups (mothers with children, single mothers), and presence of chronic conditions. In addition, these challenges had considerable psychosocial impacts. However, our findings were limited in several ways. First, our snowball sampling technique may have resulted in the recruitment of participants who knew each other and shared similar experiences and opinions. For instance lack of knowledge of some community- based food programs could be a reflection of the use of snowball sampling in creating more homogeneous sample population. Second, interviews were not recorded; we relied on written notes provided by bilingual RAs. This choice may have posed a threat to the credibility of results, but was required due to the sensitive nature of the study topic, participants’ immigration status, and the need to ensure a non-threatening environment. However, RAs verified their notes with participants during and after interviews and revised them accordingly. Considering the above mentioned limitations our recommendations may only be applicable to the group sampled and other visible immigrants who may share similar challenges.

Insufficient income was identified as a major barrier to accessing sufficient, nutritious, and culturally-acceptable food. Low income has been reported consistently as a major predictor of food insecurity [13,14,16-18]. New immigrants face a confluence of factors related to inadequate income. Approximately half of our participants were unemployed or held low-paying or seasonal jobs and two-thirds received welfare. Tarasuk and Vogt [16] reported that reliance on welfare increases the likelihood of food insecurity, so strategies are needed to improve the employability of new immigrants in their own field, and to end the reliance of working adults on welfare.

Another barrier identified was the cost of housing. Housing is only considered affordable if it uses no more than one-third of household gross income [8]. Previous studies have reported that insufficient welfare and the high cost of housing contribute to food insecurity [13,15,18]. Hence, one strategy for enhancing the financial power of new immigrants may be increasing subsidized housing or public rent subsidies to allow people more financial power to acquire food.

Participants reported using resource augmentation strategies such as purchasing low-quality but affordable foods. However, they were concerned about weight gain for not only themselves but also their children. Because low quality foods often have more fat and calories, and vegetables and fruits are relatively expensive, insufficient income may lead to an increased risk for overweight/obesity and associated diseases [20]. This is of particular concern because the LA population is at increased risk for diabetes and heart disease [37].

Proficiency in English is a contributing factor to employability among recent immigrants [38]. A lack of proficiency in English or French prevented some participants from accessing meaningful employment, and also posed a major challenge when trying to communicate when purchasing food. Language barriers also affected participants’ ability to understand food labels and coupons, preventing them from making healthy choices or accessing foods at reduced prices; these findings support previous research [3,34]. It is essential to help new immigrants learn English by providing free/subsidized, comprehensive, and easily-accessible English-language programs in the community and at workplaces. Existing English language programs/services should be reviewed and evaluated to determine whether they meet the needs of new immigrants. In Canada, mean English literacy scores are significantly lower among immigrants than their native-born counterparts (about 60% of immigrants have low literacy compared with 37% of native-born Canadians) [39]. Additionally, language and literacy levels of the target population should be taken into consideration when designing food labels or providing information related to community-based resource information; information that is translated or presented at a grade 5 or lower literacy level would improve comprehensibility and help immigrants make informed decisions [40].

We observed a high degree of reliance on formal and informal assistance with money and/or food, including food banks, churches, and family/friends. However, many of these supports were limited in their ability to address the food needs of our participants. The role of food banks in alleviating food insecurity requires further exploration, because although reliance on them has increased by 26% above levels experienced before the 2008–2009 recession [41], their quality of foods and accessibility (eligibility criteria, temporal and spatial location) appears to be inadequate. Other problems with food banks include stigma, a lack of culturally-appropriate foods, limited opening hours, insufficient amount of food, and identification requirements. Previous research has also highlighted the inadequacy of food banks in addressing food insecurity [31,34]. It is crucial to make food banks more accessible to new immigrants by increasing the variety of foods offered and making them more culturally-appropriate for the communities they serve. Efforts have been made in the past few years to decrease the stigma of using food banks; the STOP Community Food Centre [42] in Toronto espouses a holistic approach, providing a variety of services including a food bank, drop-in centre, perinatal program, civic engagement, bake ovens and markets, community cooking, community advocacy, sustainable food systems education, and urban agriculture. Although this kind of program has not been systematically evaluated for its effectiveness in reducing food bank use, it provides an alternative for food insecure populations to acquire food in more dignified and socially acceptable manner. Further studies regarding the effectiveness of these kinds of programs are needed.

Other issues included cultural food preferences and the lack of culturally-appropriate resources; participants often could not access familiar foods because some of the major grocery stores and food banks did not carry these items, these foods were more expensive, or they had limited access to ethnic stores. These findings highlight the need for strategies to increase availability of culturally-appropriate food for new immigrants, e.g., making ethnic foods more available and affordable in large grocery stores.

A lack of information about food resources was another issue. Settlement agencies and community centres that serve new immigrants can play a crucial role in this aspect of information delivery. Few participants in our study mentioned the use of community or collective kitchens although there are several community kitchens in Toronto. This could be either due to the fact that our study question focused more on the barriers to accessing food, or to participants’ lack of awareness of existence of community kitchens. It would be important to promote programs such as “The Basic Shelf Experience” (used in the UK) and community kitchens including collective kitchens (offered in Canada). Both these programs not only educate and have the potential to reduce stigma; they also provide social support. These programs combine both formal and informal methods to educate immigrants about available foods, tips for effective use of food resources such as how to stretch expensive items and how to decrease fat content in food. They also facilitate dialogue around underlying health determinants such as income, social status, employment and encourage social support networks aimed at helping people cope with stress related factors [43,44]. Engler-Stringer and Berenbaum [44] found that collective kitchens in Toronto, Montreal, and Saskatoon, where participants prepared large quantities of food (more than five meals monthly), increased participants’ food security by increasing resources through cost savings, and increased access to better quality foods and variety. They were also more acceptable as they provided dignity associated with not having to access charitable resources like food banks. Participants also had reduced psychological distress. Despite its promising short-term effects, community kitchens apparently have failed to attract the most disadvantaged/ vulnerable population due to a variety of economic, psychological and structural barriers [44]. Considerable subsides may be needed for community/collective kitchens that serve those at the lowest level of income as even a small charge may be too much for families living below the poverty line. The cost of transportation could also deter participation in such programs. Further exploration will be required to tailor these types of programs to meet the specific needs of LA immigrants and to incorporate best practices that have worked in other areas. The effectiveness of community-based food programs to promote socially acceptable food acquisition and address the specific needs of the most disadvantaged/ vulnerable population requires further investigation.

Our study corroborated findings from other studies that have shown that food insecurity, which is largely due to poverty (financial constraints), has considerable physical and psychosocial impacts. The constant anxiety and stress and lowered self-esteem associated with living in poverty and being unable to provide basic needs for their families that our participants reported, has been documented by other qualitative studies [21,45]. The stigma associated with use of food banks and receiving charity led some participants to conceal their situation from their relatives and friends. These factors were confounded by others such as language difficulties and lack of interaction with others resulting in social isolation and depression [46]. Food insecurity also had some specific impacts on vulnerable groups such as people with chronic diseases who could not manage their conditions and single mothers and new mothers. All these factors underscore the urgency to address the issue of food security for new immigrants and the need to have targeted interventions for the most vulnerable.

## Competing interest

The authors declare that they have no competing interests.

## Authors’ contributions

MV contributed significantly to the conception and design of the study, training of the research assistants for interview and data collection, reviewing the transcripts, analyzing and interpreting the data, drafting the manuscript, revising and approving the final version. CD participated in reviewing the study transcripts, analyzing and interpreting the data, and reviewing and revising the manuscript for its intellectual content. Both authors read and approved the final manuscript.
